# A successful surgical repair of a gunshot injury to the left common carotid artery without neurological deficiency: A case report

**DOI:** 10.1016/j.amsu.2022.104173

**Published:** 2022-07-12

**Authors:** Abdijalil Abdullahi Ali, Abdinafic Mohamud Hussein, Hassan Kalif Abdi, Ahmed Omar Mohamed, Ali Abdulkadir Ali, Erhan Renan Ucaroglu

**Affiliations:** Department of Cardiovascular Surgery, Mogadishu, Somalia Turkey Recep Tayyip Erdogan Training and Research Hospital, Mogadishu, Somalia

**Keywords:** Penetrating neck injuries (PNI), Common carotid artery injuries (CCAI), Computed tomography angiography (CTA)

## Abstract

**Introduction and importance:**

Penetrating neck trauma is serious and has a high fatality rate, especially in individuals who suffer injuries to the common carotid artery. The mortality rates for penetrating neck trauma are estimated to be 3%–6%. Accidents that cause a lot of blood to flow, like being stabbed, shot, or hurt in a car accident, can cause a person to lose a lot of blood quickly and in a short amount of time, which can be fatal if not treated right away.

**Clinical presentation:**

we present a 26-year-old young male patient with penetrating neck trauma caused by a gunshot. The gunshot entered the right sternocleidomastoid muscle at the level of the hyoid bone and exited the left sternocleidomastoid muscle on the mid side.

**Clinical discussion:**

In a recent report on the management of major vascular injuries to the neck, carotid artery injuries accounted for about 17% of all patients presenting with penetrating neck injuries. In this case, previously published literature adds that carotid artery injury early surgical and primary repair in young patients has a good outcome.

**Conclusion:**

Considering the high morbidity and mortality associated with penetrating neck injuries, In young patients, they can be successfully managed with early surgical and primary repair with a good outcome.

## Highlights

• Mostly, carotid artery injuries are associated with neurological deficiency.
• The gold-standard approach for assessing suspected neck vascular damage is CT angiography.
• Carotid artery injury early primary surgical repair in young patients has a good outcome.

## 1 Introduction

The mortality rates for penetrating neck trauma are estimated to be 3%–6%, with the most common cause of death being massive hemorrhage from injury to vascular structures [1].

Penetrating carotid artery injuries due to knives and bullets account for 4–17% [2,3].

The standard of care for penetrating neck injuries has shifted from compulsory exploration to selected management. Surgeons are currently advocating selective operative management of patients based on comprehensive physical examination and selective diagnostic tests.

According to the anatomical level of the injury, penetrating injuries of the neck can be divided into 3 zones. Zone I extends from the clavicles and sternal notch to the level of the cricoid cartilage. Zone II is the region between the cricoid and the inferior border of the mandible. Zone III is the area between the angle of the mandible and the skull base [4].

Zone II contains the highest density of vital structures and is where penetrating injuries occur most often [5]. In this case study, we present a 26-year-old young male patient with penetrating neck trauma caused by a gunshot, successfully managed with early surgical and primary repair with a good outcome.

## 2 Case report

**Fig. 1:**
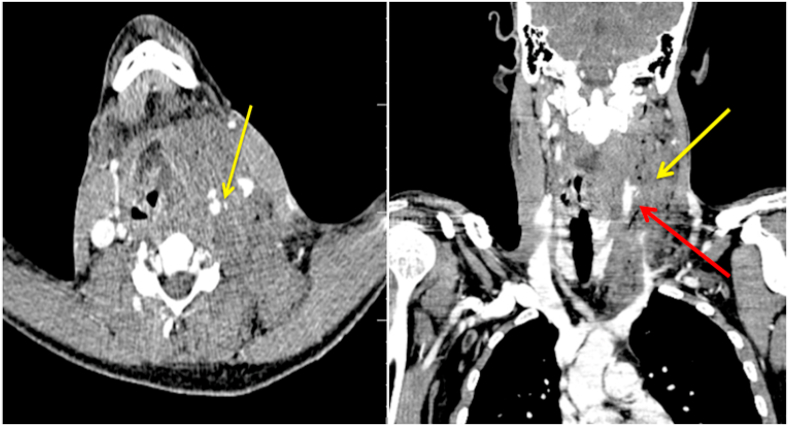
Right side CT Angiography coronal view shows Hematoma (yellow arrow), extravasation of contrast (Red arrow) and left side axial view shows extravasation of contrast and defect. (For interpretation of the references to color in this figure legend, the reader is referred to the Web version of this article.)

**Fig. 2:**
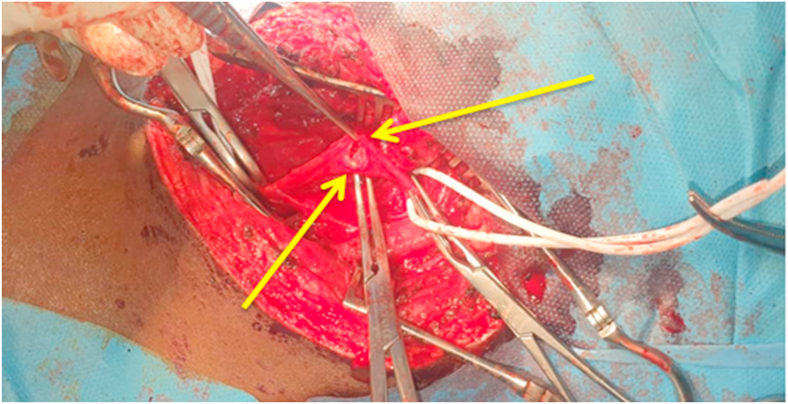
Left Common Carotid Artery Injured Area (yellow arrows). (For interpretation of the references to color in this figure legend, the reader is referred to the Web version of this article.)

A 26-year-old young male presented to our emergency department after being shot by a high-velocity bullet that entered from the right side of the neck midway through the sternocleidomastoid muscle and exited via the left side of the back just superior to the left scapula. He complained of relatively intense discomfort on the left side of his neck, as well as some swelling. No past medical or surgical history. On admission, vital signs were as follows: blood pressure = 108/76 mmHg, pulse = 84 beats/min, respiratory rate = 18 min, temperature = 37 °C, and oxygen saturation = 98% with 3L/min of oxygen given via nasal prongs. When we examined him at our hospital 3 h after the injury, we found a young man who was fully conscious, alert and with no signs of blood loss. All of the systems appeared to be completely normal. A neurological test showed normal pupils that responded to light normally. The evaluation of the cranial nerves was unremarkable. Reflexes and power in all four limbs were normal. A normal tone was discovered during a digital rectal examination. Laboratory tests were as follows: WBC = 9.17x10/mm3, Hg = 11.8 g/dL, Hct = 34.9%, PLT = 221, APTT = 28.2sec, INR = 1.37, Creatinine = 0.74 mg/dL, urea = 14, AST = 28, ALT = 10, Na = 142 mEq/L, and K = 3.7 mEq/L A significant hematoma on the left side of the cervical arteries was discovered on computed tomography angiography, with contrast extravasation most likely due to a lesion of the left proximal common carotid artery (Fig. 1). A significant hematoma on the left side of the cervical arteries was discovered on computed tomography angiography, with contrast extravasation most likely due to a lesion of the left proximal common carotid artery (Fig. 1). The patient was sent to the operating room after being diagnosed with a common carotid artery injury. The patient was administered 1500 IU of tetanus antitoxin intramuscularly and 2 g of cezol (Cefazolin) intravenously. Under general anesthesia, an oblique incision along the anterior border of the left sternocleidomastoid muscle was used to expose the common carotid artery during an emergency surgical exploratory. 5,000 units of heparin were given intravenously before vascular clamps were used. The internal and external carotid arteries were both assessed and clamped distal to the gunshot injury area. A high-velocity gunshot injury went all the way through the common carotid artery from side to side, just near where the carotid bifurcates into two. (Fig. 2). The proximal common carotid artery was in good condition, and there were no macroscopic findings of heat injury in the distal carotid arteries.

**Fig. 3:**
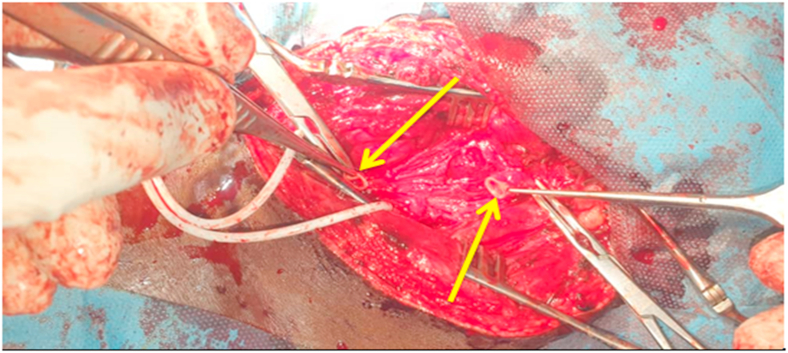
After resection of injured part proximally and distally of the left common carotid artery.

**Fig. 4:**
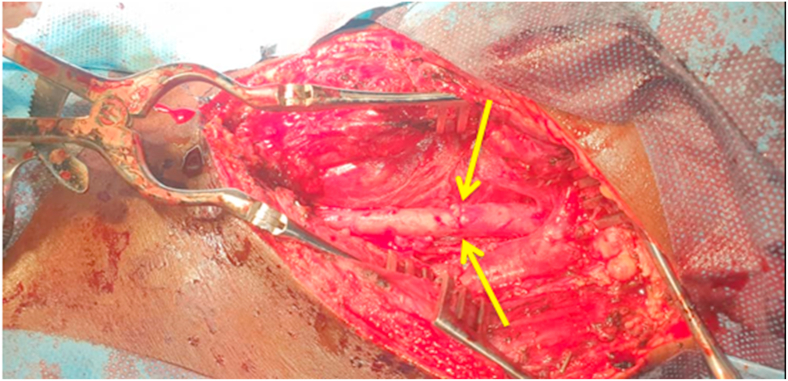
End-End anastomosis of left common carotid artery proximally and distally.

In our surgical technique, a common carotid artery damage site was resected proximally and distally (Fig. 3). Then, we preferred a primary end-to-end anastomosis (Fig. 4). A continuous 6/0 Prolene suture was used to fix the damage. The wound was irrigated with normal saline with rifampicin. The wound edge was sutured and a drainage tube was placed. Postoperatively, the patient had no neurological deficits after surgery. On the eighth day after surgery, the patient was discharged with aspirin 100 mg and no complications. The patient has been followed up with at the Cardiovascular Surgery clinic on a monthly basis for the last year and is still symptom-free, with a repeat duplex scan of the carotids coming back normal.

## 3 Discussion

Injuries to the carotid artery occur in approximately 6% of penetrating neck trauma and account for 22% of all penetrating cervical vascular injuries [6].

The patients are mostly young, and despite the low incidence, mortality and morbidity are very high. Mortality is, in most series, between 5% and 40%, and persistent neurological consequences are reported in up to 80% of patients [7].

Patients who present with "hard signs" of vascular injury such as external hemorrhage, internal hemorrhage into the trachea, esophagus, or mouth, evidence of a pulsatile or expanding hematoma in the neck, acute neurological symptoms, tracheal deviation, or elevation of the floor of the mouth from a hematoma require prompt operative intervention [8,9].

In patients with hemodynamic stability and no obvious signs of arterial or venous injury, workup with CT arteriography, duplex ultrasonography, or color flow duplex is warranted [10].

Development of modern surgical techniques, when technically feasible, for carotid artery repair has become the accepted method of treatment [11,12].

In a study by Reva et al. [13], they analyzed 46 patients with common and internal carotid artery injuries. Poor outcomes of carotid artery ligation and carotid artery repair were performed in 100% and 30%, respectively, and it is recommended that repair is the du Toit et al. [14] considered that the carotid artery should be repaired rather than ligated when technically possible, subsequent ischemic or hemorrhagic cerebral injury being the primary method of choice.

This case report contributes to our current literature on early primary surgical repair in young patients and has a good outcome when the patients come in a short period of time, approximately within 3 hours. Our case became the first in Somalia and East Africa. In developing countries, there are huge and widely limited cardiovascular surgery centers. Surgical techniques and advanced interventions, such as endovascular, were limited. The patient's vital signs were stable, and there was no neurologic damage after temporary carotid artery clamping and repair. During postoperative follow-up, color Doppler ultrasound can be performed as an examination approach. During postoperative follow-up, color Doppler ultrasonography revealed normal blood flow in this patient.

This case has been reported in line with the SCARE 2020 criteria [15].

## 4 Conclusion

As this was the first case in Somalia to be successfully managed and operated on, we used CT angiography with contrast, which is the gold standard for diagnosing suspected vascular damage, to figure out what was wrong.

Early primary surgical repair in young patients has a good outcome when the patients come in a short period of time, approximately within 3 hours.

## Ethical approval

According to our hospital rule, Ethical approval is only required in articles but not case reports.

## Sources of funding

There is no funding source for this study.

## Author contribution

All authors contributed toward writing, analysis, drafting, and revising the paper and they gave final approval of the version to be published, and agree to be accountable for all aspects of the work.

## Registration of research studies

1. Name of the registry: Not applicable.

2. Unique Identifying number or registration ID: Not applicable.

3. Hyperlink to your specific registration (must be publicly accessible and will be checked): Not applicable.

## Consent

Written informed consent was obtained from the patient for publication of this case report and accompanying images.

## Guarantor

Abdijalil Abdullahi Ali.

## Declaration of competing interest

I declare that there is no competing interest related to the study, authors, other individuals, or organizations.
